# Exosomes Secreted from CXCR4 Overexpressing Mesenchymal Stem Cells Promote Cardioprotection via Akt Signaling Pathway following Myocardial Infarction

**DOI:** 10.1155/2015/659890

**Published:** 2015-05-05

**Authors:** Kai Kang, Ruilian Ma, Wenfeng Cai, Wei Huang, Christian Paul, Jialiang Liang, Yuhua Wang, Tiejun Zhao, Ha Won Kim, Meifeng Xu, Ronald W. Millard, Zhili Wen, Yigang Wang

**Affiliations:** ^1^Department of Pathology and Laboratory Medicine, College of Medicine, University of Cincinnati Medical Center, Cincinnati, OH 45267, USA; ^2^Department of Cardiology, The People's Hospital of Sanya, Sanya, Hainan 572000, China; ^3^Department of Urinary Surgery, Xining City Hospital, Qinghai 810000, China; ^4^Department of Pharmacology and Cell Biophysics, College of Medicine, University of Cincinnati Medical Center, Cincinnati, OH 45267, USA; ^5^Infection Hospital of Nanchang University, Nanchang, Jiangxi 330002, China

## Abstract

*Background and Objective.* Exosomes secreted from mesenchymal stem cells (MSC) have demonstrated cardioprotective effects. This study examined the role of exosomes derived from MSC overexpressing CXCR4 for recovery of cardiac functions after myocardial infarction (MI). *Methods. In vitro*, exosomes from MSC transduced with lentiviral CXCR4 (Exo^CR4^) encoding a silencing sequence or null vector were isolated and characterized by transmission electron microscopy and dynamic light scattering. Gene expression was then analyzed by qPCR and Western blotting. Cytoprotective effects on cardiomyocytes were evaluated and effects of exosomes on angiogenesis analyzed. *In vivo*, an exosome-pretreated MSC-sheet was implanted into a region of scarred myocardium in a rat MI model. Angiogenesis, infarct size, and cardiac functions were then analyzed. *Results. In vitro*, Exo^CR4^ significantly upregulated *IGF-1α and pAkt levels and downregulated active caspase 3 level* in cardiomyocytes. Exo^CR4^ also enhanced VEGF expression and vessel formation. However, effects of Exo^CR4^ were abolished by an Akt inhibitor or CXCR4 knockdown. *In vivo*, Exo^CR4^ treated MSC-sheet implantation promoted cardiac functional restoration by increasing angiogenesis, reducing infarct size, and improving cardiac remodeling. *Conclusions.* This study reveals a novel role of exosomes derived from MSC^CR4^ and highlights a new mechanism of intercellular mediation of stem cells for MI treatment.

## 1. Introduction

Myocardial infarction (MI), resulting from the interruption of blood supply to the heart, is a primary target for experimental stem/progenitor cell-based therapies. Although the therapeutic effect of mesenchymal stem cells (MSC) has been attributed to their differentiation into reparative or replacement cell types [1], the therapeutic importance of cardiovascular lineage remains to be elucidated. CXCR4, a G-protein-coupled 7-transmembrane receptor, in conjunction with its primary ligand stromal cell-derived factor- (SDF-) 1*α* serves as a major regulator of stem/progenitor cell activities. The importance of SDF-1*α*/CXCR4 signaling has been documented in CXCR4 knockout rats, which die in utero. This indicates a fundamental developmental role for this receptor-ligand axis [2]. In previous studies from our laboratory, MSC were genetically engineered using* ex vivo* adenoviral transduction to overexpress CXCR4 in MSC (MSC^CR4^) [3]. Those studies determined that MSC^CR4^ secreted multiple cytokines such as vascular endothelial growth factor (VEGF) and insulin-like growth factor- (IGF-) 1*α* in response to hypoxia, augmenting endogenous regenerative processes [3].

Exosomes are naturally occurring membrane-bound nanovesicles (50–100 nm) which play a role in the selective release of membrane or cytosolic proteins, RNAs, and/or microRNAs (miRNAs), mediating some aspects of cell-to-cell signaling [4]. However, generally speaking these exosomes are not technically “responsible for the selective release” of signaling molecules, as they do not “select” the molecular cargo themselves; they just act as transporters. Recently, A2B1 (hnRNPA2B1) has been proved to artificially load selected small regulatory RNAs into exosomes through recognition of specific short motifs [5]. Exosomes are formed in intracellular vesicular bodies of most cells and released from the cell when multivesicular bodies fuse with the plasma membrane [6]. Previous studies indicated that exosomes released from progenitor cells contain secreting paracrine factors that stimulate neovascularization, thereby mediating cardiac protection during myocardial ischemia/reperfusion injury [7]. The current study is focused on determining the effects of employing a cell-patch containing isolated and purified exosomes from MSC^CR4^ in a rat model of myocardial infarction (MI). Patches were implanted into the infarcted zone, and multiple techniques were used to study the engraftment levels and changes in blood vessel formation. This study then used those results to investigate the mechanistic participation of exosomes on the effects of MSC^CR4^ after MI and to derive any expression changes in proangiogenic factors including VEGF and IFG-1*α*. The hypothesis was that exosomes derived from Exo^CR4^ would release their angiogenic factors into the implanted cell sheet, leading to restoration of cardiac functions through angiogenesis, both inside the myocardial infarct region and outside the epicardium (cell sheet).

## 2. Material and Methods

### 2.1. Isolation and Culture of Rat Bone Marrow MSC and Neonatal Cardiomyocytes (CM)

Experiments using animals or animal-derived materials were conducted in accordance with the Guide for the Care and Use of Laboratory Animals (NIH Publication number 85-23, revised in 1996) and under guidelines and protocols approved by the University of Cincinnati Institutional Animal Care and Use Committee. MSC from healthy 8-week-old Sprague-Dawley (SD) rats were isolated from bone marrow aspirates as described previously [3, 8]. Passage 2–4 MSC were used in the study. MSC were cultured in high glucose Dulbecco's modified Eagle's medium (DMEM) containing 10% fetal bovine serum (FBS) and 1% antibiotics (streptomycin and penicillin) at 37°C in humid air with 5% CO_2_. When the cell population reached 70–80% confluence, all cell experiments were performed without serum or antibiotics and repeated at least three times. Neonatal CM was isolated from ventricles of 2-day-old neonatal rats using neonatal CM isolation kit (Worthington Biochemical) as described [9]. The cells were maintained in Dulbecco's modified Eagle's medium (DMEM medium, Hyclone) with 10% FBS.

### 2.2. Transfection of MSC with Lentiviral Constructs

Lentiviral vector backbone pGreenPuro shRNA (pGP) was purchased from System Biosciences (Mountain View, CA). The multiple cloning site of pGP between BamHI and EcoRI restriction enzyme sites was replaced with the siRNA template designed by us to construct the shRNA vector pGP-siR per manufacturer's protocol. The lentiviral vector overexpressing CXCR4 was described in our previous publication [10]. To produce lentivirus for use in MSC transfection, 293NT producer cells (System Biosciences) were transiently transfected with pGP-siR and pGP-null by pPACKH1 HIV Lentivector Packaging Kit (System Biosciences). Viral supernatant was harvested and added to the host cell for 72 hours for viral genome integration into MSC genome. MSC in this study were divided into the following groups: (1) MSC transfected with null lentivirus vector (MSC^Ctrl^); (2) MSC transfected with lentiviral vector to overexpress CXCR4 (MSC^CR4^); (3) MSC transfected with siRNA against CXCR4 (MSC^siCR4^) to knock down CXCR4 expression.

### 2.3. Exosome Isolation from Conditional Medium

All cells were grown in Roswell Park Memorial Institute (RPMI) medium supplemented with 5% of exosome-depleted FBS (dFBS) (Systems Biosciences) and 1% penicillin/streptomycin (Invitrogen). MSC were seeded in 100 mm cell culture dish at 2 × 10^5^ cells/dish. After allowing cells to attach overnight, the medium was replaced and the cells were cultured for 3 days in the fresh medium for approximately 80% confluence. Exosomes were subsequently isolated from conditioned medium using ExoQuick-TC exosome purify reagent (Systems Biosciences) as manufacturer's protocol. The resulting exosome pellets were resuspended in approximately 200 *μ*L PBS and stored at −80°C for subsequent quantification (using Bio-Rad Protein Assay Dye Reagent) and for molecular analysis. Exosomes in this study were divided into the following groups: (1) exosomes isolated from MSC^Ctrl^ (Exo^Ctrl^); (2) exosomes isolated from MSC^CR4^ (Exo^CR4^); (3) exosomes isolated from MSC^siCR4^ (Exo^siCR4^).

### 2.4. Transmission Electron Microscopy (TEM) Assay

Exosome pellet was fixed by 2% glutaraldehyde and 2% paraformaldehyde (PFA) in 0.1 M Sorensen's phosphate buffer for 3 hours as described [10]. After fixation in 1% osmium tetroxide (OsO_4_) for 30 min, exosomes were washed and dehydrated in graded series of 50%, 70%, 80%, 90%, and 100% ethanol and embedded in Epon 812. Ultrathin sections (~60 nm) were cut with a Leica Ultracut UCT ultramicrotome, stained with uranyl acetate and Reynolds lead citrate, and viewed with a transmission electron microscope (JEOL JEM-1230).

### 2.5. Internalization of Exosomes

Exosomes were prelabeled with PHK67 (Sigma) as described [11] and then cocultured with CM in DMEM containing 10% FBS for 48 hours. The uptake of exosomes by CM was stopped by washing in cold PBS, followed by fixation in 4% PFA for determination of MSC exosome-transduced CM using Olympus BX41 microscope equipped with CCD (MagnaFire, Olympus) camera.

### 2.6. TUNEL Assay

CM treated with various exosomes were exposed to a hypoxic condition (O_2_/CO_2_ incubator-MCO-18M, Sanyo) at 37°C with 1% O_2_ : 5% CO_2_ : 94% N_2_ for 24 hours. Then the cells from various treatment groups were fixed with 4% paraformaldehyde for 30 minutes at room temperature. TUNEL was performed using DeadEnd Fluorometric TUNEL System (Promega) as per instructions of the manufacturer. Nuclei were stained with DAPI for 5 minutes at room temperature. The numbers of TUNEL positive nuclei were determined after counting the cells in randomly selected microscopic fields at 20x using fluorescence microscope (BX41, Olympus). Images were recorded using a digital camera and analyzed using MagnaFire 2.1 software.

### 2.7. Tube Formation Assay

The assay was performed with a tube formation assay kit (Chemicon) according to the manufacturer's instructions [12]. Briefly, the formation of capillary-like structures was assessed in a 24-well plate using growth factor-reduced matrigel. Human umbilical vein endothelial cells (HUVEC) (3 × 10^4^ cells/well) were seeded on the top of solidified matrigel (500 *μ*L/well) 2 hr. prior to exosomes (100 *μ*L/well) treatment. The HUVEC were treated as following groups: (1) HUVEC + Exo^Ctrl^, HUVEC treated with Exo^Ctrl^; (2) HUVEC + Exo^CR4^, HUVEC treated with Exo^CR4^ (3) HUVEC + Exo^siCR4^, HUVEC treated with Exo^siCR4^; (4) HUVEC + Exo^Ctrl^ + LY, HUVEC + Exo^Ctrl^ group treated with additional PI3K inhibitor LY294002 (10 *μ*M); (5) HUVEC + Exo^CR4^ + LY, HUVEC + Exo^CR4^ group treated with additional LY294002 (10 *μ*M). After 16 hr., the total tube length was quantified using software Image-Pro Plus 5.1. LY294002 was purchased from Cell Signaling Technology.

### 2.8. Western Blotting

Briefly, MSC and derived exosomes were lysed in lysis buffer, pH 7.4 (50 mM HEPES, 5 mM EDTA, and 50 mM NaCl), 1% Triton X-100, protease inhibitors (10 *μ*g/mL aprotinin, 1 mM phenylmethylsulfonyl fluoride, and 10 *μ*g/mL leupeptin), and phosphatase inhibitors (50 mM sodium fluoride, 1 mM sodium orthovanadate, and 10 mM sodium pyrophosphate). The protein samples (40 *μ*g) were electrophoresed using SDS-polyacrylamide gel and electroimmunoblotted as described [8]. After protein transfer, the samples on PVDF membranes were visualized using an enhanced chemiluminescence system (Invitrogen), exposed to X-ray film, and then quantified by laser scanning densitometry. The specific antibodies used for detection of antigens of interest are listed in the following: anti-CXCR4 (Millipore), anti-Akt, anti-pAkt (Cell Signal Technology), anti-IGF-1*α*, anti-CD9, anti-CD63, and anti-*β*-actin antibody (Santa Cruz Biotechnology).

### 2.9. Quantitative RT-PCR (qPCR)

The cells were collected for total RNA extraction using TRIzol reagent (Life Technologies). Total RNA was used for reverse transcription in a miScript II RT kit (Qiagen). The primer sequences used were listed as follows: VEGF forward primer 5′-GGCAGCTTGAGTTAAACGAAC-3′, reverse primer 5′-TGGTTGGTGTGA-CATGGTTAATCGGTC-3′; *β*-actin forward primer 5′-TGTCATCCTCCCAATCCCTCAGAA-3′, reverse primer 5′-TGTGGTGCCAGATCTTCTCCATGT-3. The fold changes of each target mRNA expression relative to *β*-actin were calculated based on the threshold cycle (C_T_) as *r* = 2^−Δ(ΔC_T_)^, where ΔC_T_ = C_T_ (target) − C_T_ (*β*-actin) and Δ(ΔC_T_) = ΔC_T_ (experimental) −  ΔC_T_ (control). A Bio-Rad CFX96 Real-Time System and calculations were performed using Microsoft Excel.

### 2.10. Cell Sheet Preparation

MSC (3.2 × 10^5^ cells/cm^2^) treated with exosomes from either MSC^Ctrl^ or MSC^CR4^ were seeded onto 3.5 cm temperature-responsive dishes purchased from CellSeed Inc. (Tokyo, Japan) and cultured cells with 15% FBS-DMEM at 37°C. Then, confluent cells on the temperature-responsive dishes were incubated at 20°C. By 10–20 min, cells detached spontaneously and floated up into the medium as monolayered cell sheet. Immediately after detachment, cell sheet was transferred onto the infarcted area of the myocardium as described [13, 14].

### 2.11. Surgical Procedures for Myocardial Infarction

Ligation of the left anterior descending artery (LAD) was performed in SD female rats (200–250 g), as previously described [12]. Briefly, isoflurane anesthesia was induced by spontaneous inhalation and maintained under general anesthesia with 1-2% isoflurane. The inhalation gas was a mixture of air and oxygen and isoflurane. The animals were mechanically ventilated using a rodent ventilator (Model 683, Harvard Apparatus, South Natick, MA) connected to a tracheal tube. Body temperature was maintained at 37°C throughout the surgical procedure. The heart was exposed by left side limited thoracotomy and LAD was ligated with a 6-0 polyester suture 1 mm from tip of the normally positioned left auricle. Before closing the thoracic cavity, positive end-expiratory pressure was applied to inflate the lungs fully; then, muscle layers and skin were closed separately. This model provides accurate and reproducible information about infarct size. To investigate the role of exosome in heart functions, SD rats were randomly divided into the following groups: (1) sham group: sham operated rats had a loose suture placed around the LAD coronary artery; (2) MI group (MI alone created by LAD ligation); (3) MI + PBS group (MI + Phosphate buffered saline alone treatment); (4) MI + MSC group (MI + ordinary MSC sheet); (5) MI + MSC^ExoCtrl^ group (MI + exosomes isolated from MSC transfected with null lentivirus vector, then treated with MSC sheet); (6) MI + MSC^ExoCR4^ group (MI + exosomes isolated from MSC transfected with CXCR4 over-expressing lentivirus vector and then treated with MSC sheet); (7) MI + MSC^ExosiCR4^ group (MI + exosomes isolated from MSC transfected with siRNA against CXCR4 lentivirus vector and then treated with MSC sheet).

### 2.12. Immunohistochemical Analysis

The vascularization was identified by von Willebrand factor (vWF) expression [12]. Briefly, heart tissues at 4 weeks after MI were harvested, fixed in 10% formalin, and sectioned at 5 *μ*m thickness. Cardiac troponin T (cTnT) was used to identify CM. Signals were visualized with secondary antibodies conjugated to donkey anti-rabbit (FITC; Jackson ImmunoResearch) or donkey anti-mouse (Tritc; Jackson ImmunoResearch), respectively. DAPI was used to identify nuclei. Fluorescent imaging was performed with Olympus BX41 microscope and vessel numbers per mm^2^ were counted in 4 randomly selected fields.

### 2.13. Analysis of Left Ventricular (LV) Fibrosis

Fixed heart tissues were embedded in paraffin, and LV cross-sections from mid-LV to apex stained with Masson trichrome were used to quantify fibrosis in the left ventricle in various treatment groups. The LV area images on each slide were filmed by using an Olympus BX41 microscope equipped with CCD (MagnaFire, Olympus) camera. LV fibrosis area and total LV area of each image were measured using the Image-Pro Plus (Media Cybernetics Inc., Carlsbad, CA, USA), and the fibrosis area as a percentage of the total LV area was calculated as (fibrosis area/total LV area) × 100%.

### 2.14. Cardiac Function Assessment by Echocardiography

Echocardiography (iE33 Ultrasound System, Phillips) with a 15 MHz probe was performed 4 weeks after cell sheet placement, blinded to the study group or treatment, to assess systolic, diastolic dimensions. Hearts were imaged in 2D long-axis view at the level of the greatest LV diameter with animals under light general anesthesia. LV end-diastolic and end-systolic diameters were measured from M-mode recordings using the leading-edge method. LV ejection fraction (EF) was calculated as EF(%) = [left ventricular end-diastolic dimension (LVDd)^3^ − left ventricular end systolic dimension (LVDs)^3^]/(LVDd)^3^  × 100%. Fractional shortening (FS) at the level of the MI and patch application was also determined as [(LVDd − LVDs)/LVDd] × 100%. All measurements were performed according to the American Society for Echocardiography leading-edge technique and averaged over three consecutive cardiac cycles.

### 2.15. Statistical Analysis

Results were statistically analyzed with the use of the StatView 5.0 software package (Abacus Concepts Inc., Berkeley, CA). All values were expressed as mean ± SEM. Comparison between groups for echocardiographic longitudinal data was performed by two-way analysis of variance (ANOVA) for repeated measures. All resulting differences were analyzed by one-way ANOVA, followed by a Bonferroni/Dunn test or unpaired *t*-test. *P* value <0.05 was considered statistically significant.

## 3. Results

### 3.1. Isolation and Identification of the MSC-Secreted Exosomes

**Figure 1:**
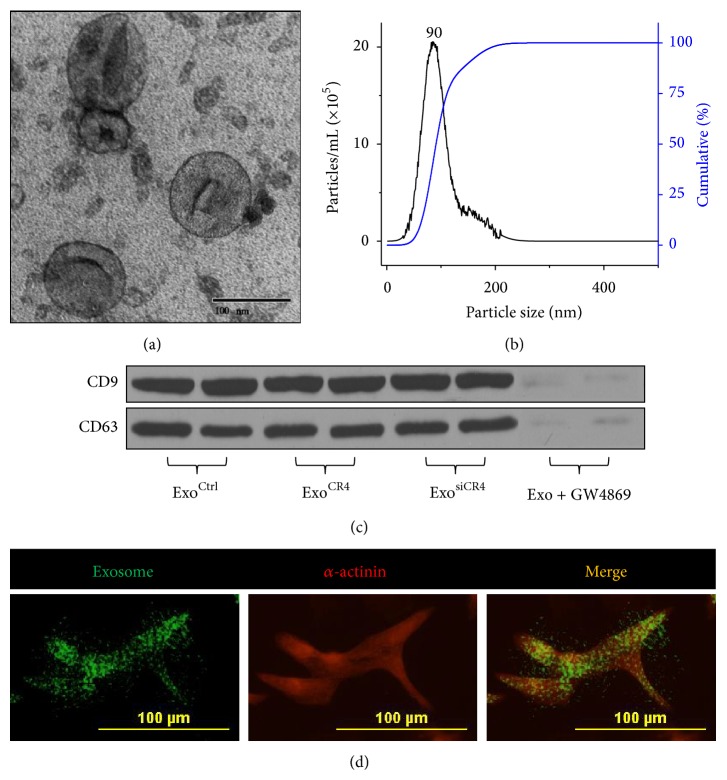
Characterization of MSC-derived exosomes. (a) Transmission electron microscopy (TEM) micrograph of exosomes purified from MSC. Isolated exosomes showed spherical and membrane encapsulated with the diameters varying between 40 and 90 nm. (b) Representative dynamic light scattering (DLS) number distribution measurement of isolated exosome population demonstrates a single peak (~90 nm diameter) indicating they are free of contamination. (c) Expression of specific exosomal markers, CD9 and CD63, in exosomes was identified by Western blotting. Exo^Ctrl^, exosomes isolated from MSC transfected with null lentivirus (MSC^Ctrl^); Exo^CR4^, exosomes isolated from MSC transfected with lentivirus overexpressing CXCR4 (MSC^CR4^); Exo^siCR4^, exosomes isolated from MSC transfected with lentivirus siRNA against CXCR4 gene (MSC^siCR4^); Exo + GW4860, exosomes isolated from MSC with additional sphingomyelinase inhibitor GW4860. (d) Uptake of exosomes by neonatal cardiomyocytes (CM). CM were stained by cardiac specific antibody, sarcomeric *α*-actinin (red). MSC^CR4^-derived exosomes were labeled with PKH67 (green) and incubated with CM for 48 hr.

Putative exosome fractions from conditioned media of MSC were isolated to investigate the paracrine effect of MSC via exosome release. High resolution transmission electron micrographs showed that MSC-derived exosomes exhibited rounded and double-membrane structures with a size of approximately 40–90 nm (Figure 1(a)), which is similar to previous descriptions [6, 7]. DLS analysis was performed to define the size of these exosomes. The size distribution profile displayed a bell-shaped curve, suggesting a physically homogeneous population with a peak at 90 nm (Figure 1(b)). Western blot analysis confirmed that exosome fraction expressed CD9 and CD63, both well-established exosome markers [4] (Figure 1(c)). In contrast, the exosome release from MSC was blocked by sphingomyelinase inhibitor GW4869 pretreatment, as indicated by significant downregulation of CD9 and CD63 expression levels. Exosomes were directly labeled with PKH67 (green fluorescence) and then cocultured with CM to determine whether the secreted exosomes were incorporated into recipient cells. Immunocytochemical analysis demonstrated that PKH67 (green color) was colocalized in the most sarcomeric *α*-actinin positive CM (red color) after 48 hr. of coculture (Figure 1(d)).

### 3.2. MSC Released CXCR4-Enriched Exosomes

**Figure 2:**
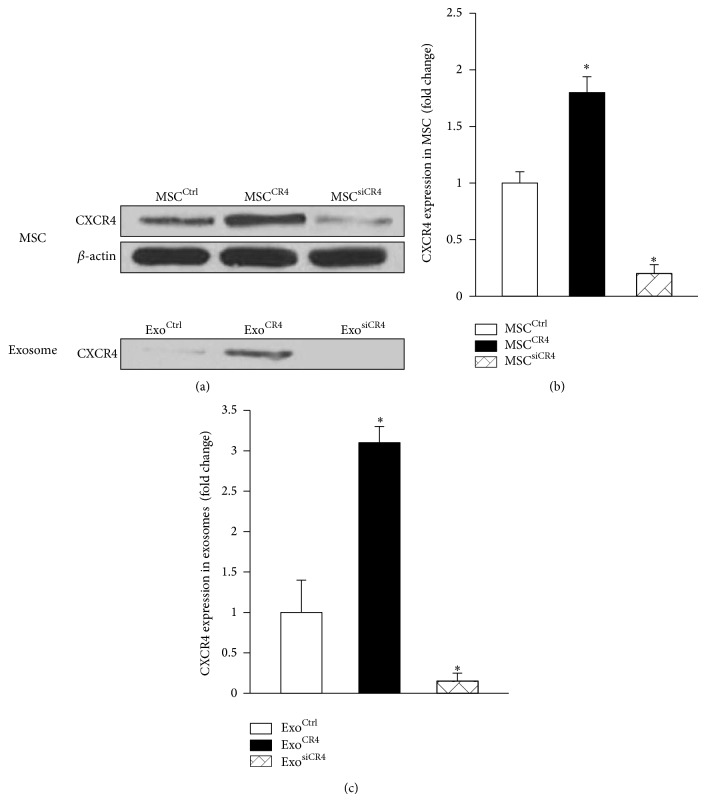
Expression of CXCR4 in MSC and MSC-derived exosomes. (a) CXCR4 expression was identified by Western blotting in MSC from various groups with quantitative analysis. ((b) and (c)) The expression of CXCR4 in MSC-derived exosome was detected by Western blotting and quantitative analysis. All values expressed as mean ± SEM. ^*^ *P* < 0.05 versus MSC^ctrl^ or Exo^Ctrl^, *n* = 4 for each group.

In order to assess whether MSC-derived exosomes possess CXCR4 and provide an alternative CXCR4 protein delivery system, lentivirus conveying control vector, CXCR4 overexpressing vector, and CXCR4-siRNA were constructed as confirmed by Western blotting (Figure 2(a)). Exosomes were then isolated from cell culture mediums of various MSC lines to identify the cargo loading CXCR4. Western blots revealed that the exosomes derived from MSC^CR4^ (Exo^CR4^) exhibited a significantly higher CXCR4 level than MSC^Ctrl^-derived exosomes (Exo^Ctrl^) (Figure 2(b)). However, with siRNA against CXCR4 in MSC (MSC^siCR4^), the CXCR4 level was significantly downregulated, leading to a significant decrease CXCR4 level in exosomes (Exo^siCR4^) as compared with other groups (Figure 2(b)).

### 3.3. The Antiapoptosis Effect of CXCR4-Enriched Exosomes on CM via Activating PI3K/Akt-Associated Signaling Pathways

**Figure 3:**
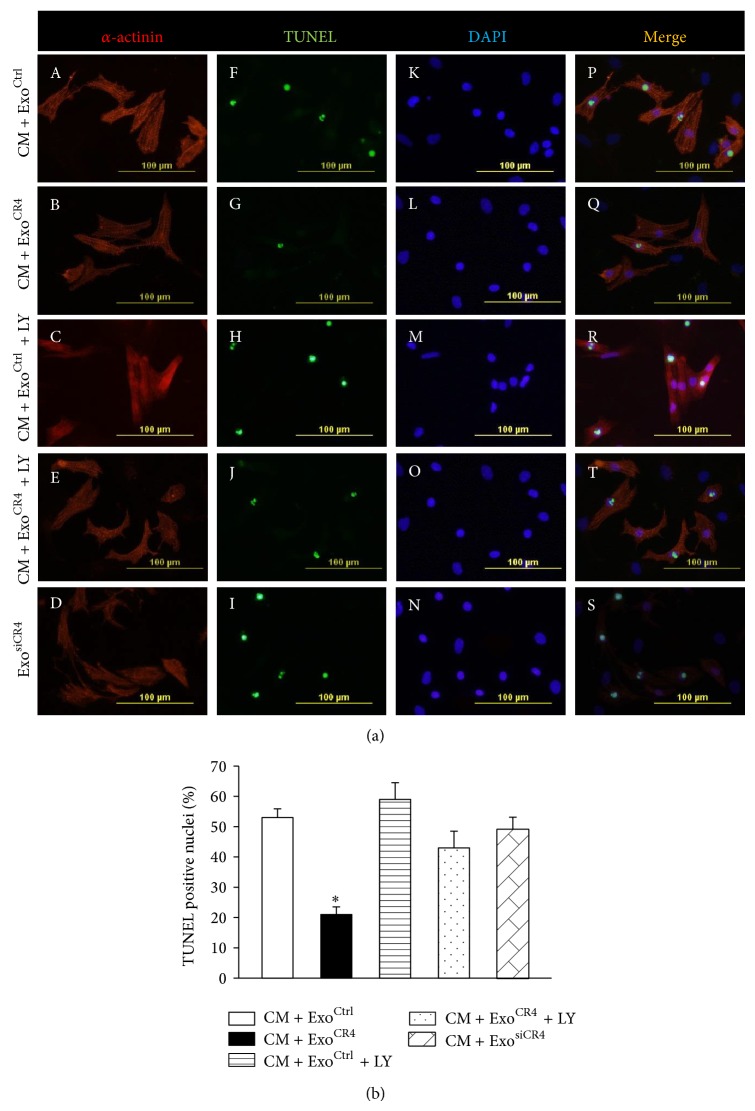
The effect of MSC^CR4^-derived exosomes on apoptosis. (a) TUNEL staining for CM apoptosis under hypoxic condition. CM labeled with cardiac specific antibody, sarcomeric *α*-actinin (red). Nuclei were labeled with DAPI (blue) and TUNEL positive nuclei (green). Original magnification ×200. (b) Quantitative data for the numbers of TUNEL positive nuclei in various experimental groups. All values expressed as mean ± SEM. ^*^ *P* < 0.05 versus Exo^Ctrl^; *n* = 6 for each group. CM + Exo^Ctrl^, CM treated with MSC^Ctrl^-derived exosomes; CM + Exo^CR4^, CM treated with MSC^CR4^-derived exosomes; CM + Exo^Ctrl^ + LY, CM + Exo^Ctrl^-treated with LY294002 (a PI3K inhibitor); CM + Exo^CR4^ + LY, CM + Exo^CR4^ treated with LY294002; CM + Exo^siCR4^, CM treated with MSC^siCR4^-derived exosomes.

In order to assess the functional transfer of CXCR4 by exosomes and the potential to correct the various hypoxia-induced cascades and prevent occurrence of apoptosis, rat CM with exosomes derived from MSC^Ctrl^, MSC^CR4^, or MSC^siCR4^ were treated in hypoxic conditions to explore whether exosomes facilitate the uptake of therapeutic proteins into injured cells that contribute to cardiac protection. TUNEL analysis showed the number of apoptotic cells (green) was significantly decreased in Exo^CR4^ treated CM (CM + Exo^CR4^ group, 21.3% ± 1.3) as compared to Exo^Ctrl^ treated CM (CM + Exo^CR4^ group, 53.6% ± 2.7; Figures 3(a) and 3(b)). The antiapoptotic effects of CXCR4 were abolished, as evidenced by increased TUNEL positive CM number in CM + Exo^CR4^ + LY (PI3K inhibitor LY294002) group as compared with CM + Exo^CR4^ group (Figure 3). The effects of CXCR4-enriched exosomes were completely abolished by siRNA targeting CXCR4 (62.4% ± 2.9, Figures 3(a) and 3(b)). The IGF-1*α*/PI3K/Akt pathway was assessed to elucidate the potential mechanisms responsible for enhanced survival rate of Exo^CR4^ treated CM under hypoxic conditions.

**Figure 4:**
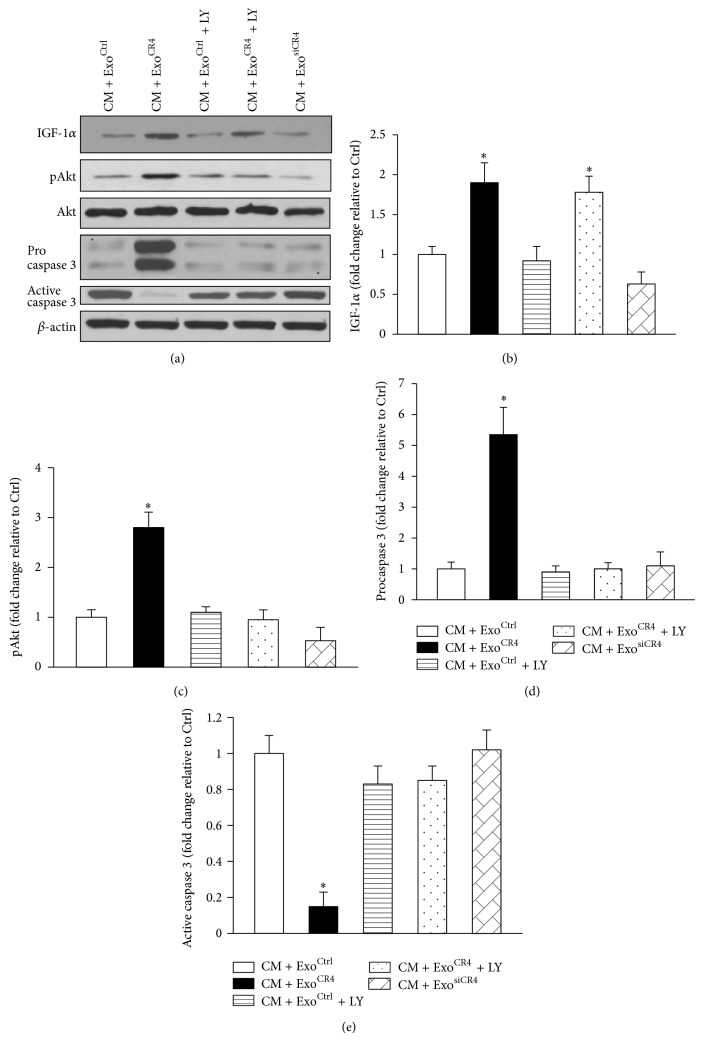
The expression of Akt signaling pathway related genes in CM with various exosome treatments. (a) The expression level of pAkt, IGF-l*α*, procaspase 3, and active caspase 3 in CM was analyzed by Western blotting. ((b)–(e)) Quantitative data of pAkt, IGF-l*α* procaspase 3, and active caspase 3 in CM. ^*^ *P* < 0.05 versus CM + Exo^ctrl^. All values expressed as mean ± SEM, *n* = 6 for each group.

IGF-1*α* and phosphorylation of Akt (pAkt) as well as procaspase 3 levels dramatically increased in CM + Exo^CR4^ group as compared to CM + Exo^Ctrl^ group and CM + Exo^siCR4^ group, whereas the level of active caspase 3 was significantly downregulated (Figure 4(a)). In contrast, with the selective PI3K inhibitor LY294002 (LY), activated PI3K/Akt pathway induced by Exo^CR4^ was inhibited as indicated by significantly decreased expression of pAkt and procaspase 3 and increased expression of active caspase 3, as compared to CM + Exo^CR4^ (Figures 4(c), 4(d), and 4(e)). However, no significant difference was observed among CM + Exo^Ctrl^, CM + Exo^siCR4^, CM + Exo^Ctrl^ + LY, and CM + Exo^CR4^ + LY groups. Interestingly, LY294002 had no effect on IGF-1*α* expression (Figure 4(b)).

### 3.4. Effect of Exo^CR4^ on Tube Formation in HUVEC

**Figure 5:**
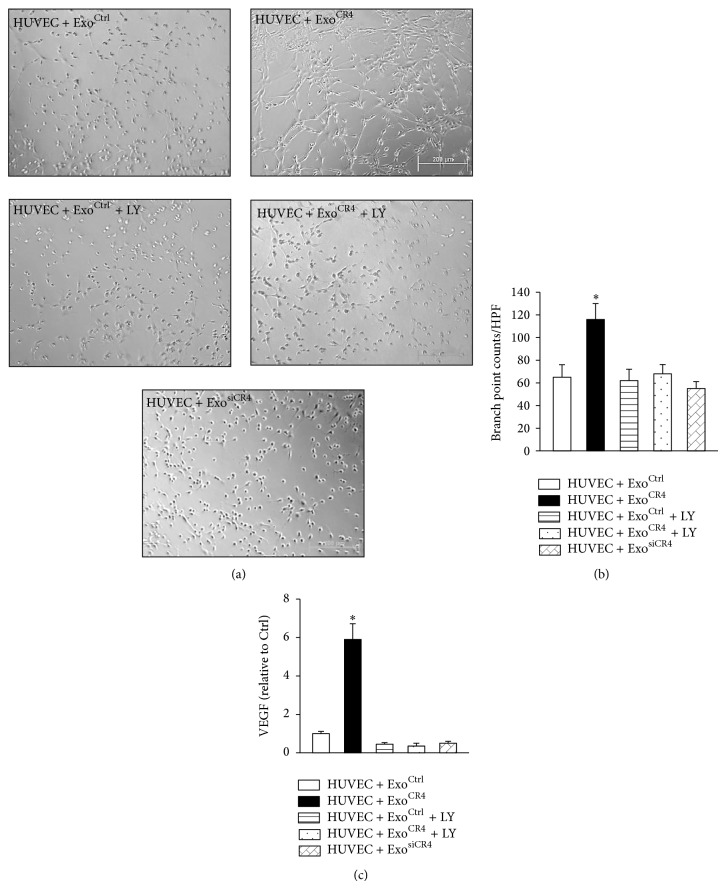
The* in vitro* angiogenic properties of MSC^CR4^-derived exosomes. (a) Induction of tube formation by MSC-derived exosomes in HUVEC. (b) Quantitative data of total branch points in various groups. (c) VEGF expression in various groups was analyzed by qPCR. ^*^ *P* < 0.05 versus Exo^Ctrl^. All values expressed as mean ± SEM, *n* = 6 for each group. HUVEC + Exo^Ctrl^, HUVEC treated with MSC^Ctrl^-derived exosomes; HUVEC + Exo^CR4^, HUVEC treated with MSC^CR4^-derived exosomes; HUVEC + Exo^Ctrl^ + LY, HUVEC + Exo^Ctrl^ treated with LY294002 (a PI3K inhibitor); HUVEC + Exo^CR4^ + LY, HUVEC + Exo^CR4^ treated with LY294002; HUVEC + Exo^siCR4^, HUVEC treated with MSC^siCR4^-derived exosomes.

Tube formation assay was performed using HUVEC to evaluate proangiogenic effects of CXCR4-enriched exosomes* in vitro*. Specifically, HUVEC in the control treatment group (HUVEC + Exo^Ctrl^) exhibited small round shapes and did not spread. However, when HUVEC were treated with Exo^CR4^ for 16 hr., they started migration and alignment, followed by the development of capillary tubes, sprouting of new capillaries, and finally formation of the cellular networks (Figures 5(a) and 5(b)). These cells became elongated, forming thing cords of interconnecting cells, and branching to form a network of capillary-like structures. The number of tube-like structures was significantly increased in HUVEC + Exo^CR4^ group as compared to HUVEC + Exo^Ctrl^ or HUVEC + Exo^siCR4^ group, or any other groups (Figure 5(b)), accompanied with significant upregulation of VEGF level (Figure 5(c)). However, the proangiogenic effect of Exo^CR4^ was attenuated by LY294002 treatment (HUVEC + Exo^CR4^ + LY) as indicated by downregulation of VEGF level as well as significantly reduced tube-like structure number as compared to Exo^CR4^ treated group.

### 3.5. Exo^CR4^ Promotes Angiogenesis and Reduces Infarction Size

**Figure 6:**
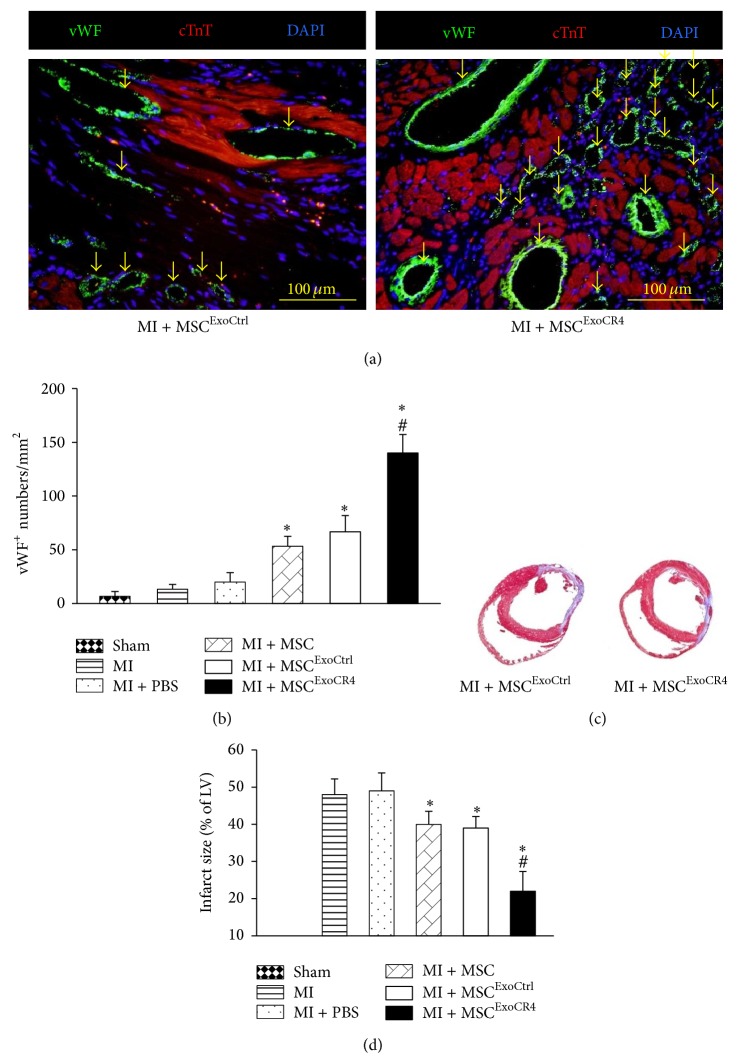
Effects of exosome pretreated MSC patch on* in vivo* angiogenesis in infarcted myocardium. (a) The vessel formation in the infarcted border area was identified by vWF (green color, yellow arrows). Cardiomyocytes were identified by cardiac troponin T (cTnT) and all nuclei were stained with DAPI. Scale bar = 100 *µ*m. Original magnification ×400. (b) Quantitative data for vessel density in treatment groups. ^*^ *P* < 0.05 versus MI group; ^#^ *P* < 0.05 versus MI + MSC^ExoCtrl^ group. (c) Masson's trichrome staining and quantitative data for fibrotic size in various treatments. (d) The percentage of infarct size in hearts of various groups. ^*^ *P* < 0.05 versus MI; ^#^ *P* < 0.05 versus MI + MSC^ExoCtrl^ group. Sham: sham operated rats had a loose suture placed around the LAD coronary artery; MI (myocardial infarction alone created by LAD ligation); MI + PBS (MI + PBS alone treatment); MI + MSC (MI + MSC transplantation); MI + MSC^ExoCtrl^ (MI + Exo^Ctrl^ pretreated MSC transplantation); MI + MSC^ExosiCR4^ (MI + Exo^siCR4^ pretreated MSC); MI + MSC^ExoCR4^ (MI + Exo^CR4^ pretreated MSC). ^*^ *P* < 0.05 versus MI; ^#^ *P* < 0.05 versus MI + MSC. All values expressed as mean ± SEM, *n* = 6 for each group.

To verify whether CXCR4-enriched exosomes play a role in angiogenesis for the repair of infarcted heart* in vivo*, we implanted cell patch system pretreated with exosomes from various groups. At 4 weeks after exosome pretreated cell patch graft, we assessed angiogenesis in the infarcted heart using immunostaining for von Willebrand factor (vWF) (Figure 6(a)). Quantitative data showed that the number of vWF positive vessels was markedly increased in Exo^CR4^ pretreated group (MI + MSC^ExoCR4^) as compared to control group (Figure 6(b)). Additionally, the infarction size was significantly reduced in MI + MSC, MI + MSC^ExoCtrl^, MI + MSC^ExosiCR4^, and MI + MSC^ExoCR4^ groups as compared to MI group or MI + PBS group, but MI + MSC^ExoCR4^ group was the lowest as assessed by Masson's trichrome staining (Figures 6(c) and 6(d)).

### 3.6. Assessment of Cardiac Functions

**Figure 7:**
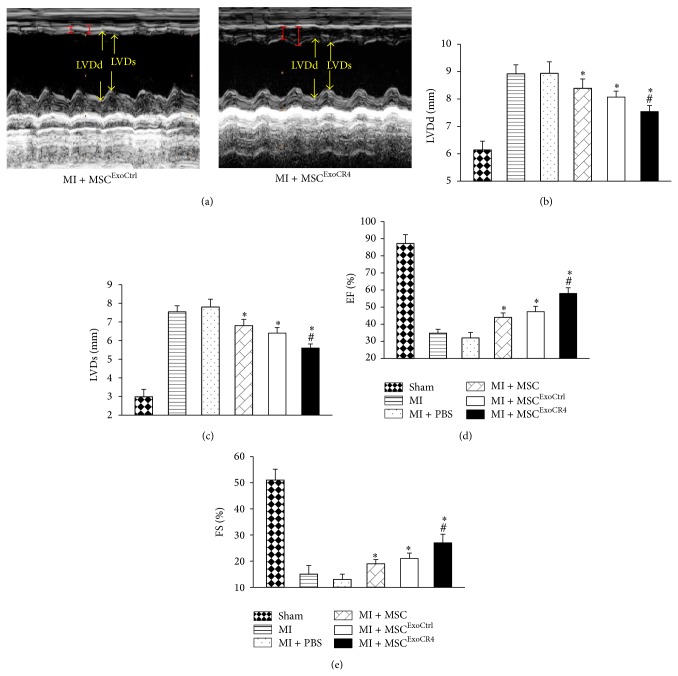
Cardiac function assessed by echocardiography at 4 weeks after cell patch graft. (a) M-mode echocardiography data in various experimental groups. ((b)–(e)) Quantification analysis for LVDd, LVDs, EF, and FS. LVDd: left ventricular diastolic left ventricular diameter, LVDs: systolic left ventricular diameter, EF: left ventricular ejection fraction, and FS: left ventricular fraction shortening. ^#^ *P* < 0.05 versus MI + MSC^ExoCtrl^ group. ^*^ *P* < 0.05 versus MI; ^#^ *P* < 0.05 versus MI + MSC. All values expressed as mean ± SEM, *n* = 6 for each group.

In order to gain an understanding of whether MSC sheet pretreated with CXCR4-enriched exosomes contributes to functional recovery of ischemic heart* in vivo*, cardiac functions (LVDd, LVDs, EF, and FS) were evaluated by echocardiography. Although the LV cavity (LVDd and LVDs) was dilated 4 weeks after cell sheet implantation after MI (Figure 7(a)) in comparison to sham group, the MI + MSC or MI + MSC^ExoCtrl^ group, or MI + MSC^ExosiCR4^ group, or MI + MSC^ExoCR4^ group exhibited a significantly reduced LV remodeling, and MI + MSC^ExoCR4^ group exhibited the most significantly reduced LV remodeling as compared to MI or MI + PBS group, as shown by decreased LVDd and LVDs (Figures 7(b) and 7(c)). Furthermore, EF and FS were significantly higher in MI + MSC and MI + MSC^ExoCtrl^ groups, but most significantly increased in the MI + MSC^ExoCR4^ group as compared to MI or MI + PBS group (Figures 7(d) and 7(e)).

## 4. Discussion

Cell-based therapy has emerged as a promising strategy for the treatment of ischemic heart diseases via myocardial repair. The paracrine effects of exosomes introduce an alternative solution to the therapeutic applications of MSC in regenerative medicine [4, 15]. Indeed, exosomes can achieve some beneficial effects through the usage of small soluble biological compounds such as growth factors, chemokines, cytokines, transcription factors, genes, and RNAs [15]. However, the delivery of these factors to the target cells with stable integrity and biological potency remains a challenge. As a bilipid membrane vesicle, exosomes not only have the capacity to carry a large cargo load, but also protect the contents such as protein and RNA from degradation by enzymes or chemicals [14, 15]. In recent years, exosomes have also been implicated in cell communication or pathogenesis. For example, exosomes secreted by cardiomyocyte progenitor cells were reported to stimulate the migration of the endothelial cells [16].

**Figure 8:**
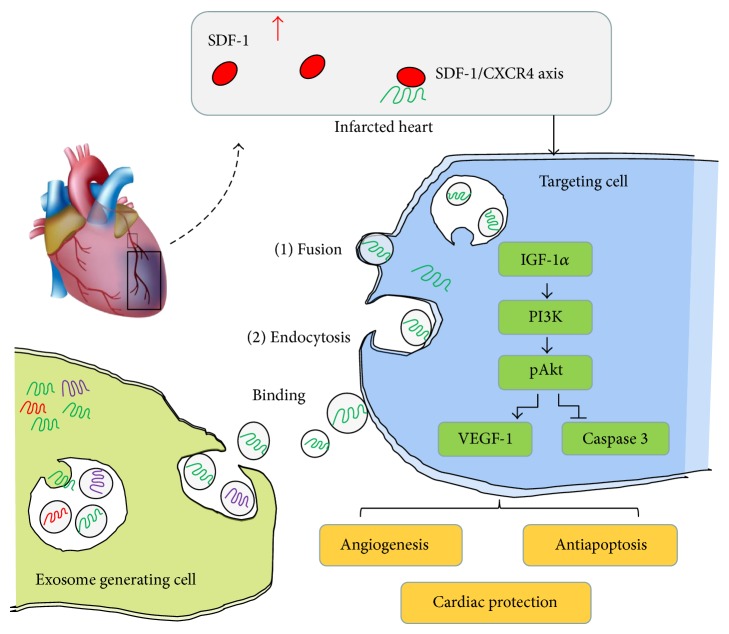
Schematic depiction of functional exosomes secreted from MSC overexpressing CXCR4 for activation of signaling pathways for restoration of LV function after MI. In exosome generating cells, CXCR4 are selectively incorporated into the intraluminal vesicles (IVLs) of multivesicular endosomes (MVEs) from the plasma membrane. Then, by fusing with the plasma membrane, MVEs release CXCR4-enriched exosome into the extracellular milieu. CXCR4-enriched exosomes can bind to the plasma membrane of the targeting cells. These recruited CXCR4-enriched exosomes may either fuse directly with the plasma membrane or fuse with delimiting membrane of an endocytic compartment. Then, released CXCR4 can be delivered into the membrane or cytosol of the targeting cell which contributes to SDF-1*α*/CXCR4 interaction. In infarcted heart tissue, increased binding of SDF-1*α* to CXCR4 activates G-protein-coupled receptor kinases, which activate a cascade of signaling pathways in cells. Pretreatment of stem/progenitor cells with CXCR4-enriched exosome can reduce MI-induced cell death and promote angiogenesis through PI3K/Akt signaling pathway activation, thereby enhancing heart function improvement.

The potential role of CXCR4-enriched exosomes was demonstrated for the repair of the infarcted heart (Figure 8). The* in vitro* studies showed that CXCR4-enriched exosomes released by MSC^CR4^ were transferred to cardiomyocytes, leading to an increase in cardiomyocyte survival under hypoxic conditions. This was also associated PI3K/Akt signaling pathway activation.* In vivo* studies demonstrated that cell sheets pretreated with CXCR4-enriched exosomes promoted angiogenesis and demonstrated subsequent cardiac function improvement. Evidence was also provided showing that MSC^CR4^ enhances the release of proangiogenic factors and promotes MSC endothelial differentiation under hypoxic conditions and that cardiac functions improved when MSC^CR4^ cell patches were implanted into myocardial ischemic hearts [17]. A study by Lai et al. also showed that exosomes secreted by MSC were able to reduce myocardial ischemia/reperfusion injury [18], which supports the results of this study. Purified exosomes from bone marrow-derived MSC were isolated using differential centrifugation and ExoQuick precipitation to investigate the mechanisms of paracrine activities of exosomes from CXCR4 overexpressing MSC. It was found that MSC-derived exosomes had phenotypes similar to those derived from other sources [7, 14]. Interestingly, the expression level of CXCR4 was significantly higher both in MSC^CR4^ and Exo^CR4^ over those of MSC^Ctrl^ and MSC^siCR4^, suggesting that the level of CXCR4 in exosomes can be enhanced by overexpressing CXCR4 in MSC.

CXCR4 is a G-protein-coupled receptor and can activate several G-protein-mediated downstream signaling pathways after stimulation [2, 19]. This is a critical factor involved in homing, endothelial cell migration, and engraftment of hematopoietic stem and progenitor cells [2]. One endpoint of CXCR4 signaling is the activation of transcription factors [19]. In addition, the SDF-1*α*/CXCR4 axis was reported to be critical for activation of PI3K in ischemic cardiomyocytes, thereby mediating acute cardioprotection [19]. Furthermore, TUNEL assay revealed that apoptosis in cardiomyocytes treated with MSC^CR4^-derived exosomes was significantly decreased as compared to control group, whereas this protective effect was abolished by PI3K/Akt inhibitor, LY294002, which led us to hypothesize that MSC^CR4^-derived exosomes could promote cardioprotection through the Akt signaling pathway. Expression of key downstream molecules of the Akt signaling pathway was examined by Western blot to test this hypothesis. VEGF signaling pathway plays an essential role in the vascular homeostasis and the angiogenic cascade [20]. IGF-1*α* itself is a somewhat less efficacious inducer of angiogenesis as compared with other angiogenic growth factors such as VEGF. The antiapoptosis activity of IGF-1*α* has been attributed to downregulation of cleaved caspase-3 [21]. Caspase 3 is a member of the cysteine-aspartic acid protease (caspase) family and interacts with caspase 8 and caspase 9. Sequential activation of caspase plays a central role in the execution phase of cell apoptosis [22]. It is well proved that MSC^CR4^-derived exosomes activate a series of downstream growth factors, such as IGF-1*α* and Akt. Cells were treated with LY294002, a PI3k/Akt inhibitor, to determine paracrine effects of MSC^CR4^-derived exosomes which were mediated through Akt signaling. Although LY294002 had no effect on IGF-1*α* expression, the antiapoptosis effect as well as tube formation induced from MSC^CR4^-derived exosomes was markedly abolished. These results provided further evidence supporting our hypothesis that Akt plays a key role in angiogenic processes. We also observed that exosomes from MSC^CR4^ deleting CXCR4 genes by siRNA did not show cardiac protection effects* in vitro* as well as functional recovery of the infarcted heart* in vivo* (data not shown), indicating that CXCR4 plays critical role in stem cell functions.

There are several issues to be addressed before exosome-based therapy is widely used in clinical practice. Therefore, further efforts need to (1) identify tissue specific exosome subpopulation; (2) engineer or modify exosome surface antigen and internal content to incorporate large amounts of cardioprotective-proteins or microRNAs (miRNAs); (3) discover the mechanisms of cell uptake; (4) develop efficiency of targeting strategies; (5) develop cost effective methods to generate optimum amount patient-derived exosomes.

The results from* in vitro* studies showing the proangiogenic effects of MSC^CR4^-derived exosomes were further confirmed by* in vivo* MI model with cell patch system. Our data indicate that cardiac functions were improved by transplantation of exosome-treated MSC via increased angiogenesis in the infarcted heart. Moreover, exosomes from MSC overexpressing CXCR4 showed better efficiency for reducing left ventricular remodeling and promoting restoration of heart function after MI, confirming that CXCR4 is a key factor for angiogenesis and cell survival. Taken together, this study demonstrated that overexpression of CXCR4 in MSC would be an effective strategy to enhance the release of exosomes containing cardioprotective factors.

## 5. Conclusion

CXCR4-enriched exosomes can be acquired from CXCR4 overexpressing MSC, and treatment with these exosomes can protect cardiomyocytes from ischemic injury both* in vitro* and* in vivo*. This novel role of CXCR4-enriched exosomes on cardiac protection highlights a new perspective into intercellular mediation of tissue injury after MI. Upregulation of the Akt signaling pathway contributed to these beneficial effects, suggesting that CXCR4-enriched exosomes may serve as an additional therapeutic strategy to promote cell survival and angiogenesis in ischemic hearts, potentially engendering a novel approach to the development of biologics for repair of MI.
